# Supplementary material for: Chronological Gene Expression of Human Gingival Fibroblasts with Low Reactive Level Laser (LLL) Irradiation
Source: J Clin Med. 2021 May 1;10(9):1952. doi: 10.3390/jcm10091952 (PMC8125544; doi:10.3390/jcm10091952)
Supplement: Supplementary file 1 [file jcm-10-01952-s001.zip › Additional data 1.pdf]

Additional data 1

DEGs of the up-regulated genes at 1 hour after LLL irradiation.

Gene Symbol	Fold Change	p-value	Gene Symbol	Fold Change	p-value
SNAR-G1	2.39	4.00.E-04	ZNF432	1.75	2.98.E-02
TRPM5	2.34	9.70.E-03	MIR637; DAPK3	1.73	2.58.E-02
MIR4277	2.22	2.21.E-02	LOC100506257	1.73	3.87.E-02
POLR2J3; POLR2J2	2.17	1.00.E-04	LOC105375666	1.72	2.80.E-03
GSTA2	2.12	8.00.E-04	MIR521-2	1.71	3.28.E-02
HCP5	2.11	1.62.E-02	FAM87B	1.7	1.00.E-04
MIR3135B	2.01	3.70.E-03	MSTO1	1.69	3.55.E-02
SNORD18C; RPL4	2.01	2.11.E-02	TEX38	1.67	4.16.E-02
MIR338	2	9.00.E-03	MIR3130-2	1.67	4.44.E-02
LOC100506123	1.97	1.11.E-02	LOC101926940	1.67	1.40.E-02
TSSK3	1.93	3.20.E-03	LOC102724087	1.67	4.51.E-02
MIR4756	1.88	3.57.E-02	NPIPB6	1.67	7.70.E-03
GTF2IP20	1.88	2.47.E-02	LOC105376834	1.66	2.10.E-03
ZNF557	1.88	4.64.E-02	LOC105373547; LOC105373552	1.65	5.00.E-03
LOC101929787	1.87	3.80.E-03	FAM185A	1.65	3.93.E-02
IGLC2; IGLJ3	1.87	6.60.E-03	IGHV1OR21-1	1.65	8.20.E-03
SCARNA20	1.85	2.35.E-02	LOC105369295	1.64	2.85.E-02
DCHS1	1.83	1.86.E-02	IGLV2-18	1.62	4.00.E-04
POMZP3	1.83	8.20.E-03	OXT	1.62	2.02.E-02
ZBTB8A	1.79	4.00.E-04	PARTICL	1.62	2.73.E-02
WASIR2	1.79	1.06.E-02	PRELID3A	1.61	2.90.E-03
LOC105379221	1.78	1.20.E-02	SNHG12	1.61	1.11.E-02
LOC101930275	1.77	1.90.E-03	LINC00633	1.61	4.64.E-02
ZNF730	1.75	3.36.E-02	SLC25A5-AS1	1.6	1.29.E-02
DNAAF3	1.75	1.13.E-02	KRT14	1.6	2.20.E-02
