# Supplementary material for: Chronological Gene Expression of Human Gingival Fibroblasts with Low Reactive Level Laser (LLL) Irradiation
Source: J Clin Med. 2021 May 1;10(9):1952. doi: 10.3390/jcm10091952 (PMC8125544; doi:10.3390/jcm10091952)
Supplement: Supplementary file 1 [file jcm-10-01952-s001.zip › Additional data 2.pdf]

Additional data 2

DEGs of the down-regulated genes at 1 hour after LLL irradiation.

Gene Symbol	Fold Change	p-value	Gene Symbol	Fold Change	p-value
SNORD13P3	-2.3	4.19.E-02	LOC105373174	-1.61	9.50.E-03
ZNF138	-2.16	1.42.E-02	RPS15A	-1.6	5.60.E-03
MIR125B1	-2.04	4.60.E-02	OR2A1	-1.59	2.88.E-02
RPL23AP32	-2.03	2.82.E-02	MIR520A	-1.58	4.00.E-03
ALPPL2	-1.9	1.96.E-02	HOXB13	-1.57	1.80.E-02
MIR4279	-1.88	4.90.E-02	NRARP	-1.57	3.46.E-02
LOC102724913	-1.88	2.70.E-02	SNORD4B; RPL23A	-1.57	5.90.E-03
CYP4F30P	-1.86	1.20.E-03	PABPC1L2B-AS1	-1.56	2.40.E-03
SNORD126	-1.81	2.55.E-02	MIR3188	-1.56	3.54.E-02
MIR3163	-1.8	1.33.E-02	MIR329-2	-1.55	4.10.E-02
RAB6C	-1.79	1.48.E-02	SNORD114-17	-1.55	2.79.E-02
RNVU1-6	-1.75	1.44.E-02	LOC105377378	-1.54	7.60.E-03
MIR1321	-1.75	1.20.E-02	SCN4B	-1.54	1.98.E-02
SNORA36B; MIR664A	-1.7	7.00.E-03	MIR128-2	-1.54	4.10.E-03
IGKV2D-24	-1.67	3.54.E-02	LOC105376517	-1.53	3.94.E-02
IGLV2-11	-1.65	1.94.E-02	LOC101928919	-1.53	3.11.E-02
RAC3	-1.65	2.21.E-02	LOC105375599	-1.53	1.00.E-02
SNORD114-28	-1.64	1.81.E-02	LOC105374160	-1.53	2.00.E-02
SNORA80A	-1.64	4.31.E-02	E2F3	-1.52	1.37.E-02
SCARNA5	-1.62	3.70.E-02	SLC35G3	-1.51	3.48.E-02
CGB2	-1.62	2.03.E-02	LSP1P3	-1.51	9.00.E-04
HIST1H2AM; HIST1H3J	-1.62	4.08.E-02	HOXB9	-1.5	3.06.E-02
FBXW4P1	-1.62	2.20.E-02	SNORD18B; RPL4	-1.5	3.64.E-02
LOC100506217	-1.61	1.55.E-02	LOC105371452	-1.5	4.69.E-02
FAM26F	-1.61	3.89.E-02	PGA5	-1.5	4.71.E-02
