# Supplementary material for: Chronological Gene Expression of Human Gingival Fibroblasts with Low Reactive Level Laser (LLL) Irradiation
Source: J Clin Med. 2021 May 1;10(9):1952. doi: 10.3390/jcm10091952 (PMC8125544; doi:10.3390/jcm10091952)
Supplement: Supplementary file 1 [file jcm-10-01952-s001.zip › Additional data 3.pdf]

Additional data 3

DEGs of the up-regulated genes at 3 hours after LLL irradiation.

Gene Symbol	Fold Change	p-value	Gene Symbol	Fold Change	p-value
SNORA74B	2.18	3.70.E-02	LOC100129476	1.6	8.70.E-03
OR10Q1	2.15	1.40.E-03	SLC6A18	1.59	7.20.E-03
MIR874	2.13	2.60.E-03	LOC105370053	1.58	2.37.E-02
MIR3665	2.03	1.98.E-02	LOC399886	1.57	2.20.E-03
LOC105377562	1.99	8.00.E-04	TPSB2; TPSAB1	1.57	3.08.E-02
TRBV5-4	1.88	4.12.E-02	BHLHE23	1.57	2.86.E-02
LOC105372327	1.81	3.70.E-03	TRAJ14	1.56	5.10.E-03
MIR4481	1.79	2.30.E-02	MOGAT3	1.55	1.18.E-02
TRAJ44	1.77	6.20.E-03	SLC52A3	1.55	3.26.E-02
SULT1A1	1.75	2.10.E-02	LOC646730	1.55	1.30.E-02
LOC105372587	1.75	3.26.E-02	LOC100505570	1.54	3.35.E-02
MIR129-1	1.74	2.06.E-02	MIR1911	1.54	1.01.E-02
LOC105379311	1.7	3.70.E-03	LOC101928255	1.53	2.24.E-02
CYB561D2	1.7	2.89.E-02	CYP4F3	1.53	4.60.E-02
SPATA19	1.69	1.50.E-03	LOC101927872	1.52	4.20.E-03
LOC101928836	1.68	1.08.E-02	SLC19A1	1.52	4.84.E-02
MIR4756	1.67	2.00.E-03	IGHE	1.52	4.58.E-02
CST5	1.66	1.16.E-02	TRAJ16	1.51	5.10.E-03
HTR3B	1.65	9.40.E-03	LOC283440	1.51	4.80.E-03
HP	1.63	1.16.E-02	GPR132	1.51	7.90.E-03
CYP2D6	1.62	1.21.E-02	GLRA4	1.51	2.97.E-02
MIR4289	1.62	6.00.E-04	SLC25A18	1.5	1.40.E-03
TMEM95	1.6	6.70.E-03	LOC642574	1.5	1.06.E-02
