# Supplementary material for: Chronological Gene Expression of Human Gingival Fibroblasts with Low Reactive Level Laser (LLL) Irradiation
Source: J Clin Med. 2021 May 1;10(9):1952. doi: 10.3390/jcm10091952 (PMC8125544; doi:10.3390/jcm10091952)
Supplement: Supplementary file 1 [file jcm-10-01952-s001.zip › Additional data 4.pdf]

Additional data 4

DEGs of the down-regulated genes at 3 hours after LLL irradiation.

Gene Symbol	Fold Change	p-value	Gene Symbol	Fold Change	p-value
SNORD114-25	-2.25	2.95.E-02	LOC105375358	-1.59	3.69.E-02
DIP2A-IT1	-2.21	3.06.E-02	SCARNA9	-1.59	1.04.E-02
HIST1H2AK	-2.1	2.00.E-04	TRIM61	-1.57	7.80.E-03
SNORD16; RPL4	-1.98	2.80.E-03	MMP25-AS1	-1.56	1.01.E-02
LOC728024	-1.93	1.72.E-02	LOC105377866	-1.55	5.50.E-03
SNORD4B; RPL23A	-1.85	1.53.E-02	TMEM160	-1.55	2.12.E-02
HIST1H3G	-1.83	1.70.E-03	TPI1P3	-1.54	8.10.E-03
RNU11	-1.8	3.69.E-02	KRTAP4-12	-1.54	1.56.E-02
MIR302D; MIR302B	-1.78	3.45.E-02	SNORD58C; RPL17	-1.54	2.74.E-02
SNORA36B; MIR664A	-1.74	4.70.E-02	ACOT2	-1.53	4.97.E-02
TAS2R46	-1.7	3.18.E-02	LOC101928509	-1.52	4.90.E-02
SNORD58A; RPL17	-1.68	2.54.E-02	RPS15A	-1.52	4.70.E-03
FAM72D; FAM72C	-1.67	4.00.E-04	MIR4718	-1.52	8.10.E-03
MIR3167	-1.66	4.50.E-03	TRAJ30	-1.51	2.35.E-02
PPIP5K1	-1.63	3.60.E-03	SNORD11	-1.51	1.20.E-03
MIR4678; MINPP1	-1.62	1.92.E-02	MIR519A1	-1.51	3.75.E-02
