# Supplementary material for: Chronological Gene Expression of Human Gingival Fibroblasts with Low Reactive Level Laser (LLL) Irradiation
Source: J Clin Med. 2021 May 1;10(9):1952. doi: 10.3390/jcm10091952 (PMC8125544; doi:10.3390/jcm10091952)
Supplement: Supplementary file 1 [file jcm-10-01952-s001.zip › Additional data 5.pdf]

Additional data 5

DEGs of the up-regulated genes at 6 hours after LLL irradiation.

Gene Symbol	Fold Change	p-value	Gene Symbol	Fold Change	p-value
CEP170P1	5.1	2.11.E-02	KMT2D	2.4	4.70.E-02
NAV2	4.14	4.27.E-02	NPIPB5; NPIPB4; NPIPB3	2.39	1.52.E-02
XYLT1	3.85	2.70.E-03	E2F7	2.38	2.60.E-03
MIR4655	3.56	3.83.E-02	NRGN	2.38	6.00.E-04
TNRC6C	3.52	2.42.E-02	TRBJ2-6	2.35	3.60.E-02
CHST3	3.33	1.30.E-03	CA5BP1	2.33	1.16.E-02
CEP170	3.29	4.06.E-02	FGL2	2.33	3.00.E-03
MIR4532	3.17	2.10.E-03	NPIPB3; NPIPB4; NPIPB5	2.31	4.10.E-02
TGFBI	3.02	4.00.E-02	LOC105373550	2.3	7.40.E-03
LOC105372640	2.99	1.94.E-02	STK10	2.29	9.80.E-03
ALPK2	2.93	4.35.E-02	RFPL4AL1	2.28	5.10.E-03
TSHZ3	2.8	4.86.E-02	MAP3K11	2.28	7.30.E-03
LOC100996713	2.73	6.91.E-05	LOC105372966	2.28	2.16.E-02
TBC1D2	2.71	6.30.E-03	SNORD111	2.27	2.76.E-02
LOC100996273	2.69	2.00.E-04	FAM120A	2.27	3.59.E-02
NPIPB4; NPIPB3	2.67	1.16.E-02	LOC105369628	2.26	3.63.E-02
FAM195A	2.66	2.33.E-02	LYZL4	2.25	1.24.E-02
ITGA2	2.62	3.60.E-03	NAV3	2.25	5.60.E-03
LRRC3	2.59	4.50.E-02	TGM2	2.21	4.71.E-02
PCMTD2	2.5	1.52.E-02	TPTE2P6	2.2	4.40.E-03
CD3EAP	2.44	8.00.E-04	STARD8	2.19	3.48.E-02
SLC25A6	2.44	2.07.E-05	SSBP3	2.19	1.58.E-02
LOC400927	2.43	6.00.E-04	HSD3BP4	2.18	1.19.E-02
ZNF609	2.41	2.01.E-02	LOC101928195	2.17	1.87.E-02
BACE1-AS	2.4	3.13.E-02	SEMA7A	2.16	7.49.E-06
