# Supplementary material for: Chronological Gene Expression of Human Gingival Fibroblasts with Low Reactive Level Laser (LLL) Irradiation
Source: J Clin Med. 2021 May 1;10(9):1952. doi: 10.3390/jcm10091952 (PMC8125544; doi:10.3390/jcm10091952)
Supplement: Supplementary file 1 [file jcm-10-01952-s001.zip › Additional data 6.pdf]

Additional data 6

DEGs of the down-regulated genes at 6 hours after LLL irradiation.

Gene Symbol	Fold Change	p-value	Gene Symbol	Fold Change	p-value
HIST1H2AI	-7.78	9.00.E-04	SNORD18C; RPL4	-2.72	1.94.E-02
HIST1H2BB	-7.46	1.50.E-03	BMS1P6	-2.69	1.56.E-02
HIST1H2AE	-6.98	5.30.E-03	CNKSR3	-2.64	2.00.E-04
HIST1H2BL	-6.34	4.20.E-03	LY96	-2.64	3.16.E-02
HIST1H2AM; HIST1H3J	-5.4	2.60.E-03	MIR572	-2.63	4.50.E-03
HIST2H2BE	-5.2	6.00.E-04	LOC105374160	-2.62	4.36.E-02
HIST1H4E	-4.68	9.70.E-03	HIST1H3I	-2.61	5.00.E-04
HIST1H2BI	-4.51	3.00.E-04	HIST1H1B	-2.57	2.00.E-04
HIST1H3F	-4.41	3.00.E-04	HIST1H4J; HIST1H4K	-2.56	9.50.E-03
HIST1H2BK	-4.35	1.50.E-03	MIR1913	-2.51	1.83.E-02
HIST2H2AB	-4.02	1.80.E-03	HIST1H2BH	-2.46	8.40.E-03
HIST1H3G	-3.92	2.00.E-04	PSAT1	-2.46	4.38.E-02
HIST1H1E	-3.9	6.89.E-05	OR2T34	-2.43	3.60.E-03
HIST1H3E	-3.77	4.33.E-02	LOC100506844	-2.43	2.60.E-03
LINC01085	-3.74	6.17.E-08	ZNF680	-2.41	1.73.E-02
SNORA12	-3.73	6.50.E-03	HIST1H2AG	-2.36	3.06.E-05
HIST1H1C	-3.64	3.00.E-04	GAREM1	-2.35	2.97.E-02
MAP3K8	-3.59	5.00.E-04	TMEM116	-2.34	1.60.E-03
HIST1H4D	-3.52	4.30.E-03	PRAMEF10	-2.34	2.40.E-03
MIR1224	-3.38	6.00.E-04	CCNE2	-2.33	4.00.E-04
SNORD13	-3.37	2.04.E-02	HIST2H3A; HIST2H3C	-2.32	2.20.E-03
HIST1H4L	-3.37	2.30.E-02	HIST2H3A; HIST2H3C	-2.32	2.20.E-03
SNORA60	-3.14	4.00.E-03	LOC101930131	-2.31	5.80.E-03
HIST1H4K; HIST1H4J	-2.8	1.05.E-02	MIR199A1	-2.28	1.16.E-02
POT1	-2.77	1.00.E-03	TMCO6	-2.28	3.08.E-02
