# Supplementary material for: Chronological Gene Expression of Human Gingival Fibroblasts with Low Reactive Level Laser (LLL) Irradiation
Source: J Clin Med. 2021 May 1;10(9):1952. doi: 10.3390/jcm10091952 (PMC8125544; doi:10.3390/jcm10091952)
Supplement: Supplementary file 1 [file jcm-10-01952-s001.zip › Additional data 7.pdf]

Additional data 7

DEGs of the up-regulated genes at 12 hours after LLL irradiation.

Gene Symbol	Fold Change	p-value	Gene Symbol	Fold Change	p-value
LOC105374715	2.56	8.60.E-03	SULT1B1	2.03	8.39.E-05
TRAJ59	2.47	2.80.E-03	SNORA80A	2.02	4.75.E-02
SNORD114-6	2.44	9.88.E-05	LINC01597	2.02	3.40.E-03
ADAMTS7P1	2.38	4.87.E-02	ZNF443	2	3.30.E-03
DDTL	2.35	2.66.E-02	LOC105372367	1.99	1.92.E-02
POU5F1P4	2.33	2.00.E-03	ANKRD30BP2	1.98	2.70.E-02
MIR3691	2.32	4.38.E-02	USP6NL	1.96	1.29.E-02
IGHV3-48	2.3	7.20.E-03	SLC25A52	1.96	9.10.E-03
LOC101928173	2.3	3.00.E-04	RBMY1J; RBMY2FP	1.95	1.17.E-02
MIR921	2.3	2.33.E-02	RNU6-56P	1.94	4.33.E-02
LOC729815	2.27	1.91.E-02	SLC19A1	1.94	1.20.E-03
LOC105378683	2.26	2.70.E-03	PMCHL1	1.93	3.68.E-02
GATA4	2.19	1.00.E-03	C16orf86	1.93	6.00.E-03
IGHV3-20	2.17	4.40.E-03	GPR149	1.9	2.20.E-03
IGFL1	2.17	5.90.E-03	LOC102723370	1.89	2.55.E-02
MIR3175	2.16	1.32.E-02	NBPF22P	1.88	3.42.E-02
LOC102723323	2.16	1.00.E-03	IGHA1; IGHV3-66	1.88	2.41.E-02
LOC105373185	2.14	9.50.E-03	MIR892A	1.88	9.30.E-03
FUT1	2.13	3.15.E-02	MIR504	1.87	4.56.E-02
KRTAP5-2	2.12	8.00.E-04	MIR3188	1.86	9.70.E-03
TBC1D3P5	2.12	2.00.E-04	SERPINA6	1.85	4.80.E-03
LRRC37A6P	2.09	7.00.E-04	FCGR1B	1.84	2.40.E-03
HIST1H2AJ	2.09	1.10.E-03	SIGLEC7	1.84	2.60.E-03
SLC35G5	2.06	7.00.E-04	DACT1	1.83	9.36.E-06
LOC105369301	2.05	8.00.E-04	LOC105378066	1.83	1.60.E-03
