# Supplementary material for: Chronological Gene Expression of Human Gingival Fibroblasts with Low Reactive Level Laser (LLL) Irradiation
Source: J Clin Med. 2021 May 1;10(9):1952. doi: 10.3390/jcm10091952 (PMC8125544; doi:10.3390/jcm10091952)
Supplement: Supplementary file 1 [file jcm-10-01952-s001.zip › Additional data 8.pdf]

Additional data 8

DEGs of the down-regulated genes at 12 hours after LLL irradiation.

Gene Symbol	Fold Change	p-value	Gene Symbol	Fold Change	p-value
CENPF	-3.36	2.09.E-02	GTF2IP20	-2.19	9.70.E-03
CEMIP	-3.1	7.00.E-04	MAMLD1	-2.19	2.07.E-02
MKI67	-2.98	2.11.E-02	ND1	-2.17	4.38.E-02
MRC2	-2.75	2.76.E-02	ITGA8	-2.15	1.61.E-02
KRT6B	-2.61	4.86.E-02	MIR3160-1	-2.15	3.41.E-02
TNKS	-2.6	1.20.E-03	NPIPA7; NPIPA8	-2.14	9.20.E-03
NPIPB4; NPIPB3	-2.54	1.23.E-02	SEMA3D	-2.13	1.27.E-05
SDHAP1	-2.51	8.20.E-03	MIR4681	-2.13	4.41.E-02
RNF213	-2.5	4.42.E-02	ZC3H12B	-2.13	6.15.E-05
CENPE	-2.49	2.94.E-02	MAP3K11	-2.1	4.80.E-03
ABI3BP	-2.49	3.89.E-02	ANK1	-2.1	4.50.E-03
METTL7A	-2.48	2.00.E-04	NAV2	-2.1	4.57.E-02
NBPF14; NBPF10; NBPF19	-2.44	4.20.E-03	FAM168A	-2.1	3.69.E-02
HEG1	-2.44	1.73.E-02	NIPBL	-2.1	4.32.E-02
IGLJ2	-2.38	2.20.E-03	KRT34	-2.08	3.00.E-04
CYTB	-2.38	2.20.E-02	FAM193A	-2.07	2.10.E-02
ZNF34	-2.38	3.20.E-03	NPIPB5; NPIPB4; NPIPB3	-2.07	2.53.E-02
LOC105370710; LOC105379204	-2.32	3.75.E-02	FBN2	-2.07	4.16.E-02
TRIM56	-2.32	6.60.E-03	B3GLCT	-2.06	4.83.E-02
RELN	-2.29	3.25.E-02	SNRPN; IPW	-2.06	5.10.E-03
ND5	-2.28	3.11.E-02	SSH1	-2.05	6.40.E-03
DCLK2	-2.27	2.10.E-03	EVI2B	-2.05	3.00.E-04
PTPN13	-2.26	2.20.E-03	GSTM5	-2.05	1.00.E-02
DST	-2.26	1.96.E-02	BAZ2B	-2.05	4.18.E-02
KRTAP1-5	-2.23	5.67.E-06	LACC1	-2.04	5.70.E-03
